# Predictive Factors of the Responses to Treatment of *Mycoplasma pneumoniae* Pneumonia

**DOI:** 10.3390/jcm10061154

**Published:** 2021-03-10

**Authors:** Eun Lee, Yun Young Lee

**Affiliations:** 1 Department of Pediatrics, Chonnam National University Medical School, Chonnam National University Hospital, Gwangju 61469, Korea; 2 Department of Radiology, Chonnam National University Medical School, Chonnam National University Hospital, Gwangju 61469, Korea; yunyoung0219@gmail.com

**Keywords:** *Mycoplasma pneumoniae*, pneumonia, treatment, prediction, response

## Abstract

The prevalence of refractory *Mycoplasma pneumoniae* (MP) pneumonia is increasing. The present study aimed to identify the predictive factors of responses to treatment of MP pneumonia in children. A total of 149 children were diagnosed with MP pneumonia, of whom 56 were included in the good response group, 75 children in the slow response group, and 18 children in no response or progression group. Data on the clinical, laboratory, and radiologic features were retrospectively obtained through medical chart reviews. The severity of pneumonia, based on the extent of pneumonic lesions on chest x-ray (adjusted odds ratio (aOR), 10.573; 95% confidence intervals (CIs), 2.303−48.543), and lactate dehydrogenase (LDH) levels (aOR, 1.002; 95% CIs, 1.000–1.004) at the time of admission were associated with slow response to treatment of MP pneumonia. Pleural effusion (aOR, 5.127; 95% CIs, 1.404–18.727), respiratory virus co-infection (aOR, 4.354; 95% CIs, 1.374–13.800), and higher LDH levels (aOR, 1.005; 95% CIs, 1.002–1.007) as well as MP-specific IgM titer (aOR, 1.309; 95% CIs, 1.095–1.564) were associated with no response or progression of MP pneumonia. The area under the curve for the prediction of no or poor response in MP pneumonia using pleural effusion, respiratory virus co-infection, LDH levels, and MP-specific IgM titer at the time of admission was 0.8547. This study identified the predictive factors of responses to treatment of MP pneumonia in children, which would be helpful in establishing a therapeutic plan and predicting the clinical course of MP pneumonia in children.

## 1. Introduction

*Mycoplasma pneumoniae* (MP) is one of the most common causes of community-acquired pneumonia in children, with its cyclic epidemics occurring every three to four years, depending on the geographic location [1,2]. MP can spread via infectious droplets, and the estimated incubation period is between four days and three weeks. MP is characterized by the absence of cell-wall material; hence, only a few antibiotics are effective against MP infection [1,2]. Although MP infection is considered to be a self-limiting disease in some cases, potentially severe MP pneumonia cases, characterized by poor response to the first-line therapy—which consists of a 7- to 14-day treatment of clarithromycin (10–15 mg/kg/day, 2–3 doses, orally) or a three-day treatment of azithromycin (10 mg/kg/day, once daily, orally)—and incomplete resolution of pulmonary lesions, are increasing [3]. With the increasing prevalence of macrolide-resistant MP and severe MP pneumonia [4], concerns regarding the prediction of the clinical course, treatment responses, and therapeutic strategies for MP pneumonia have been consistently raised [4,5]. However, studies on these issues are limited.

In cases of refractory MP pneumonia, usually defined as clinical and radiologic deterioration despite appropriate treatment for seven days or more [1,6,7,8], the risk of developing complications, such as persistent pulmonary atelectasis, consolidation, or post-infectious bronchiolitis obliterans, is increased [9]. In clinical situations, MP pneumonia cases, with poor response to treatments, including macrolides, floxacins, tetracyclines, or glucocorticoids, result in increased medical care costs and socioeconomic burdens. The reasons for poor response to treatment include macrolide resistance of MP, excessive immune responses, and the presence of triggering factors for an exaggerated immune response.

Early prediction of responses to the stepwise treatment and clinical courses in patients with MP pneumonia is unavoidable for the establishment of appropriate therapeutic plans and for reducing complications. Although studies identifying independent prediction biomarkers, such as interleukin-17A and interleukin (IL)-10, have been performed [7,10], studies on the predictive factors of responses to treatment, which include the clinical, radiologic, and laboratory findings at the time of admission with prediction models in children with MP pneumonia, are lacking. In the present study, we aimed to identify the discriminating factors for treatment responses in patients with MP pneumonia and suggest prediction models for predicting treatment responses.

## 2. Materials and Methods

### 2.1. Study Population

The present study retrospectively enrolled 149 previously healthy children (age less than 18 years) admitted at Chonnam National University Hospital due to MP pneumonia between May 2019 and February 2020 (Table 1). Of the 149 children, 56 (mean age, 7.0 years; standard deviation (SD), 4.7 years) were in the good response group, 75 (mean age, 5.4 years; SD, 3.2 years) were in the slow response group, and 18 patients (mean age, 5.7 years; SD, 2.1 years) were in the no response or progression group. Data on the clinical, laboratory, and radiologic features were retrospectively obtained by reviewing medical charts. The Institutional Review Board (IRB) approved this study with a waiver of informed consent (IRB no. CNUH-2019-261).

### 2.2. Stepwise Treatment Strategies in MP Pneumonia in the Present Study

All of the children admitted due to MP pneumonia were treated using a stepwise approach with consideration of the severity of pneumonia and response to treatment during the illness. Almost all of the patients (*n* = 144/149, 96.6%) were transferred from local clinics due to progression or no response to treatment of MP pneumonia. Considering the treatment provided at the local clinics, treatment strategies were established at the time of admission to our hospital. As the first-line treatment strategy, the patients were treated with macrolides, including azithromycin (mean, 4.5 days; SD, 4.1 days) or roxithromycin (mean, 3.8 days; SD, 2.8 days) for at least 3–7 days with 1–2 mg/kg/day (maximum 30 mg/dose) of intravenous methylprednisolone in severe MP pneumonia cases to decrease the excessive immune response in those with severe MP pneumonia [11,12]. If patients did not respond to the first-line therapy within 3–5 days, ciprofloxacin or tetracyclines were added to the treatment of patients with macrolide-resistant MP pneumonia. If patients did not respond to the second-line antibiotics after 3–5 days, methylprednisolone (10–15 mg/kg/day) pulse therapy was administered for 3 consecutive days.

### 2.3. Definitions

MP pneumonia was diagnosed based on the following items: (1) recently developed acute respiratory symptoms, including cough or sputum, in previously healthy children, (2) abnormal chest radiography with/without abnormal lung sounds, and (3) presence of MP detected by PCR or MP-specific IgM using a chemiluminescence immunoassay [13].

The severity of pneumonia was categorized according to the extent of pneumonia lesions on chest radiography performed at the time of admission: (1) mild, less than one-fourth of the total lung volume; (2) moderate, more than one-fourth but less than one-third of the total lung volume; and (3) severe, greater than one-third of the total lung volume.

The exact administration timing of second-line antibiotics; the administration duration of macrolides in macrolide-resistant MP pneumonia; and the starting points, dose, duration, and types of immune-modulators in severe MP pneumonia have not been established [12]. We decided to classify the study population into three groups according to the responses to the stepwise treatment strategy [12]: (1) good response group, patients who showed improvements on physical examination or chest radiography 2–3 days after the initial treatment; (2) slow response group, those who showed slight improvements on physical examination or chest radiography within 1 week of stepwise treatment but not within 2–3 days; (3) no response or progression group, those who did not show any improvement or exhibited deterioration of chest radiography or physical examination, even after 1 week of active stepwise treatment following admission to our hospital. In addition, we divided the study population into two groups, responder (the good and the slow response groups) and non-responder (the no response or progression group), to better understand the treatment responses in MP pneumonia.

### 2.4. Detection of MP and Macrolide Resistance Test

The specific IgM against MP was measured in blood samples using a LIAISON MP IgM kit (DiaSorin, Dublin, Ireland), while PCR for detection of MP was performed using sputum samples by utilizing the AmpliSens MP PCR kit (InterLabService Ltd., Moscow, Russia) at the time of admission [14]. The point mutations at both 2063 and 2064 in domain V of 23S rRNA were investigated using the GENECUBE system and GENECUBE Mycoplasma detection kit (Sin Corporation, Tokyo, Japan) [9].

### 2.5. Respiratory Viruses

Real-time multiplex PCR with an Anyplex II RV16 Detection kit (Seegene, Seoul, Korea) using nasopharyngeal swab samples was performed, which targets 16 respiratory viruses, including adenovirus, respiratory syncytial viruses A and B, rhinovirus, influenza viruses A and B, parainfluenza viruses 1–4, bocavirus, metapneumovirus, enterovirus, and corona viruses OC43, 229E, and NL63 [2].

### 2.6. Statistical Analysis

To compare the clinical, laboratory, microbiologic, and radiologic features between the three groups, analysis of variance or chi-square tests were used as appropriate for the variables. A logistic regression analysis was performed to identify the predictive factors of responses to treatment of MP pneumonia. To identify the prediction model for treatment response in patients with MP pneumonia, the receiver operating characteristic (ROC) curve was computed as a measure of discrimination for groups classified depending on treatment responses in MP pneumonia. All statistical analyses were performed using STATA statistical software (Version 14.1, Stata-Corp, College Station, TX, USA). A *p* value of <0.05 was considered significant.

## 3. Results

### 3.1. Baseline and Clinical Characteristics of the Study Population

The characteristics of the study population are shown in Table 2. The total duration of fever during the course of illness was longest in the no response or progression group (*p* < 0.001). The prevalence of oxygen requirements was highest in the no response or progression group (*p* = 0.006).

### 3.2. Comparisons of the Laboratory Findings and Microbiologic Features

The levels of white blood cells (*p* = 0.003 and trend *p* = 0.006), lactate dehydrogenase (LDH) (*p* < 0.001 and trend *p* < 0.001), and MP-specific IgM titer (*p* = 0.001 and trend *p* < 0.001) at the time of admission showed increasing trends with poorer responses to the treatment (Table 3). When the study population was divided into the responder and non-responder groups, significant differences were observed in lymphocyte (%), C-reactive protein, LDH, and MP-specific IgM titer. The prevalence of co-infection with respiratory viruses was significantly increased in the no response or progression group (*p* = 0.019) (Table 4). However, when the study population was divided into the responder and non-responder groups, there was no significant difference in the prevalence of respiratory virus co-infection.

### 3.3. Comparisons of the Radiologic Features

The prevalence of severe pneumonia, defined based on the extent of pneumonic lesions on chest radiography, and pleural effusion was significantly increased in the no response or progression group (Table 5).

### 3.4. Predictive Factors of Poor Response to Treatment of MP Pneumonia in Children

Severe pneumonia, based on the extent of pneumonic lesions on chest x-ray (adjusted odds ratio (aOR), 10.573; 95% confidence intervals (CIs), 2.303–48.543), and LDH levels (aOR, 1.002; 95% CIs, 1.000–1.004) at the time of admission were significantly associated with slow response to the stepwise treatment in MP pneumonia when the good response group was considered as the reference group (Table 6). Pleural effusion (aOR, 5.127; 95% CIs, 1.404–18.727), respiratory virus co-infection (aOR, 4.354; 95% CIs, 1.374–13.800), LDH levels (aOR, 1.005; 95% CIs, 1.002–1.007), and MP-specific IgM titer (aOR, 1.309; 95% CIs, 1.095–1.564) at the time of admission were associated with increased risk of no response or progression of MP pneumonia when the good response group was considered as the reference group. When the responder group was considered as the reference group, the duration between the onset of symptoms and admission (aOR, 1.162; 95% CIs, 1.025–1.317) was significantly longer in the non-responder group. In addition, pleural effusion (aOR, 4.792; 95% CIs, 1.549–14.820), LDH levels (aOR, 1.002; 95% CIs, 1.001–1.004), MP-specific IgM titer (aOR, 1.280; 95% CIs, 1.080–1.518), oxygen need at the time of admission (aOR, 7.628; 95% CIs, 1.764–32.986), and rhinovirus co-infection (aOR, 3.283; 95% CIs, 1.144–9.418) were significantly associated with the non-responder group.

### 3.5. Prediction Model for Response to Treatment in MP Pneumonia

**Figure 1:**
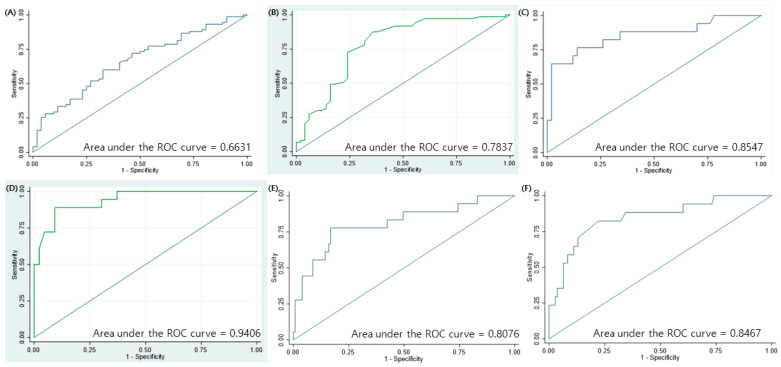
Receiver operating characteristic (ROC) curves for predicting treatment responses in children with MP pneumonia. (**A**) Area under the curve (AUC) for the slow response group based on the severity of pneumonia and LDH levels, which were significant factors associated with treatment responses in MP pneumonia in the logistic regression analysis. (**B**) AUC for the slow response group based on the age at diagnosis, severity of pneumonia, number of co-infected respiratory viruses, respiratory virus co-infection, and involvement of the right middle lobe, which were selected using the stepwise logistic regression analysis. (**C**) AUC for the no or poor response group based on pleural effusion, respiratory virus co-infection, LDH levels, and mycoplasma IgM titer at the time of admission, which were significant factors associated with treatment responses in MP pneumonia in the independent logistic regression analysis. (**D**) AUC for the no or poor response group based on severity of pneumonia, sex, and mycoplasma IgM titer at the time of admission, which were selected using the stepwise logistic regression analysis. (**E**) AUC for the non-responder group based on the duration between symptom onset and admission, pleural effusion, MP-specific IgM titer at the time of admission, oxygen need, rhinovirus co-infection, and LDH levels, which were significant factors associated with treatment responses in MP pneumonia in the logistic regression analysis. (**F**) AUC for the non-responder group using severity of pneumonia and MP-specific IgM titer at the time of admission, which were selected using the stepwise logistic regression analysis.

The area under the curve (AUC) for the prediction of slow response to the stepwise treatment in MP pneumonia according to the severity of pneumonia and LDH levels, which were significantly associated with slow response in the logistic regression analysis, was 0.6631 (Figure 1A). When stepwise logistic regression was applied to identify the appropriate model for the prediction of slow response to the stepwise treatment in MP pneumonia, the AUC was 0.7837, which included age, severity of pneumonia, respiratory virus co-infection, number of co-infected respiratory viruses, and involvement of the right middle lobe (RML) (Figure 1B). The AUC for the prediction of no response or progression in the stepwise treatment of MP pneumonia with pleural effusion, respiratory virus co-infection, LDH levels, and MP-specific IgM titer at the time of admission, which were significant factors for no response or progression group, was 0.8547 (Figure 1C). When stepwise logistic regression was applied to find the best model for the prediction of no response or progression in the stepwise treatment of MP pneumonia, the AUC was 0.9406, which included sex, severity of pneumonia, and MP-specific IgM at the time of admission (Figure 1D). When the study population was divided into the responder and non-responder groups, AUC for the non-responder group with the duration between the onset of symptoms and admission, pleural effusion, MP-specific IgM titer at the time of admission, oxygen need, rhinovirus co-infection, and LDH levels, which were identified to be significantly associated factors, was 0.8076 (Figure 1E). When stepwise logistic regression was applied to find the best model for the non-responder group, the AUC was 0.8467, which included the severity of pneumonia and MP-specific IgM titer at the time of admission (Figure 1F). The sensitivity, specificity, positive predictive value (PPV), and negative predictive values are presented in Table 7.

## 4. Discussion

In the present study, using the laboratory and radiologic findings at the time of admission, we identified the predictive factors of responses to stepwise treatment of MP pneumonia in children. More severe pneumonic involvement and higher LDH levels at the time of admission were significantly associated with a slow response to the stepwise treatment of MP pneumonia, with the good response group considered as the reference group. Pleural effusion, respiratory virus co-infection, LDH levels, and MP-specific IgM titer at the time of admission were associated with no response or progression in the stepwise treatment of MP pneumonia in children. A combination of factors, including age at the time of diagnosis of MP pneumonia, severity of pneumonia, respiratory virus co-infection, number of co-infected respiratory viruses, and involvement of RML, predicted the slow response to the stepwise treatment of MP pneumonia (AUC = 0.7837) in the stepwise logistic regression analysis. In addition, the combination of sex, MP-specific IgM titer, and severity of pneumonia at the time of admission predicted no response or progression of MP pneumonia in the stepwise treatment, with outstanding discriminatory power (AUC = 0.8406). Our results are significant because the present study has identified the predictive factors of the response to the stepwise treatment of MP pneumonia in children using diverse laboratory and radiologic features obtained at the time of admission in the era of increasing prevalence of refractory MP pneumonia. The results of the present study might be helpful in the early prediction of treatment responses, thereby establishing a therapeutic plan for refractory MP pneumonia in children.

The presence of parapneumonic effusion has been associated with longer fever duration after initiation of macrolides, regardless of macrolide resistance [15]. In our present study, fever duration during the course of illness was longer in MP patients with parapneumonic effusion than in those without parapneumonic effusion (mean ± SD, 9.5 ± 3.9 days vs. 6.1 ± 4.2 days, *p* < 0.001). Furthermore, the prevalence of parapneumonic effusion and fever duration during the course of illness were significantly increased in the no response or progression group relative to the response group. Most of the children with parapneumonic effusion (*n* = 23/24, 95.8%) in this study showed consolidation on chest radiography, and these findings were consistent with those reported in previous studies [16,17]. Although the parapneumonic effusion in all patients resolved within seven days of hospitalization, other remnant pulmonary lesions were persistent in all patients with parapneumonic effusion, despite the stepwise treatment approach; similar findings were also observed in a previous study [17]. When considering the results of the above mentioned studies [15,16,17] and the present study, the presence of parapneumonic effusion suggests exaggerated inflammatory or immune responses in children with MP pneumonia.

A previous study showed that the co-detection of viral and/or bacterial pathogens in patients with MP pneumonia has no significant impact on the clinical course and disease severity [18]. However, in our study, we identified that the prevalence of co-infection with respiratory viruses in MP pneumonia showed significantly increasing trends for poorer response to the stepwise treatment of MP pneumonia. Patients with MP pneumonia co-infected with respiratory viruses require a careful follow-up owing to the increased risk of post-infectious bronchiolitis obliterans [9], especially those with no response or progression despite the stepwise treatment; such careful follow-up is required to avoid missing the complications of MP pneumonia.

LDH, an enzyme that catalyzes the oxidative conversion of pyruvate to lactate, is a non-specific inflammatory biomarker, and elevation of LDH levels occurs in various diseases, including inflammatory diseases. LDH has been well-recognized as a good predictor of refractory MP pneumonia, with suggestions for its aid as an indicator for the use of steroid therapy in MP pneumonia [19,20], although the cut-off levels vary across studies [19,21]. The present study identified the role of LDH levels as a discriminatory factor for the response to the stepwise treatment of MP pneumonia. However, the pathophysiology underlying the association of LDH levels with refractory MP pneumonia remains unclear. Future studies are thus required to clarify the pathophysiology. In addition, diverse cytokines, such as IL-6, IL-8, IL-10, or TNF-α, can be good predictive parameters [7,10], and further studies evaluating these issues are needed in the future.

A novel chemiluminescent immunoassay system is replacing the conventional enzyme-linked immunosorbent assay (ELISA) to diagnose MP pneumonia [13]. The new chemiluminescent immunoassay is known to be quicker and have better analytic workability than the conventional ELISA [13,14,22]. Until now, studies have focused on comparing the chemiluminescent immunoassay with the conventional serologic tests for diagnosing MP pneumonia. Studies on the clinical implication of the chemiluminescent immunoassay in MP pneumonia are lacking, and the present study suggests its role at the time of admission as a predictive factor of responses to the stepwise treatment of MP pneumonia. Future studies are needed to confirm the results of the present study and identify the clinical implications of MP-specific IgM titers measured using the chemiluminescent immunoassay for detecting MP infection.

The laboratory and radiologic variables included in the AUC for the prediction of response to treatment of MP pneumonia, selected based on the results of the logistic regression analysis, were different from those selected in the stepwise logistic regression analysis used to determine the appropriate model for each group of treatment response in MP pneumonia. Severity of pneumonia was commonly involved in the prediction of slow treatment response to MP pneumonia in both the AUCs, whereas MP-specific IgM was commonly involved in the prediction of no response or progression of MP pneumonia in both the AUCs. Although the application of stepwise logistic regression can slightly increase the AUC for the prediction of responses to treatment of MP pneumonia, the application of clinical factors is also significant for the prediction of responses to treatment of MP pneumonia in children.

Our present study has some limitations. First, the present study used a small sample size and was performed in a single tertiary medical center. Therefore, the results of the present study require validation from a large-sample study. Second, patients in the good response group were older than those in the two other groups, which limits the generalization of the results. Although treatment response was determined based on the findings on chest radiography and physical examination, no response to the treatment can be somewhat subjective depending on the individual. However, timely introduction of appropriate treatment is crucial for the management of MP pneumonia in children to improve clinical outcomes [12,23]. Consensus regarding the treatment of refractory MP pneumonia has not been achieved, although various attempts have been made [12]. The stepwise treatment of MP pneumonia used in the present study was based on the reports of the concerned studies [2,12,23,24,25]. When comparing the therapeutic effects of macrolides, fluoroquinolone, or tetracyclines in MP pneumonia, regardless of macrolide resistance, the poor prognostic factors in each patient must be considered when establishing a personalized therapy for MP pneumonia in children.

## 5. Conclusions

The present study has identified predictive factors of response to treatment, classified as good response, slow response, and no response or progression, using the clinical, laboratory, and radiologic findings at the time of admission of children with MP pneumonia. The early prediction of treatment response in MP pneumonia can be helpful for the early application of effective treatment modalities and for the establishment of a therapeutic plan, especially in refractory MP pneumonia cases, thereby, improving the clinical outcomes.

## Figures and Tables

**Table 1:** Inclusion and exclusion criteria of the present study.

Inclusion	Exclusion
● Previously healthy children admitted due to MP pneumonia between May 2019 and February 2020 ● Diagnosis of MP pneumonia based on chest radiography and/or physical examination ● MP identified using the MP-specific IgM from blood samples and/or PCR for MP from sputum samples	● Children with other chronic diseases, including chronic lung diseases, neurologic diseases, or hemato-oncologic diseases

MP, Mycoplasma pneumoniae; PCR, polymerase chain reaction.

**Table 2:** Baseline and clinical characteristics of the study population according to responses to the treatment of *Mycoplasma pneumoniae* pneumonia.

Variables	Good Response	Slow Response	No Response or Progression	*p* Value *	Responder Group	*p* Value #
Baseline characteristics						
Number	56	75	18	NA	131	NA
Age at the diagnosis of MP pneumonia, mean ± SD, years	7.0 ± 4.7	5.4 ± 3.2	5.7 ± 2.1	0.045	6.0 ± 3.9	0.743
Male, *n* (%)	32/56 (57.1)	39/75 (52.0)	7/18 (38.9)	0.401	71/131 (54.2)	0.223
Presence of allergic diseases, *n* (%)	30/56 (53.6)	44/75 (58.7)	10/18 (55.6)	0.842	74/131 (56.5)	0.940
Referred cases, *n* (%)	54/26 (96.4)	72/75 (96.0)	18/18 (100.0)	0.695	126/131 (96.2)	0.399
Clinical characteristics						
Fever, *n* (%)	55/56 (98.2)	75/75 (100.0)	18/18 (100.0)	0.433	130/131 (99.2)	0.710
Duration of fever, days	6.0 ± 3.7	6.2 ± 4.3	10.4 ± 4.8	<0.001	6.1 ± 10.4	<0.001
Hemoptysis, *n* (%)	3/56 (5.4)	0/75 (0.0)	0/18 (0.0)	0.079	3/131 (2.3)	0.517
Duration between symptom onset and admission, days	6.2 ± 3.7	6.6 ± 3.8	8.6 ± 4.0	0.071	6.4 ± 3.7	0.026
Need for supplemental oxygen, *n* (%)	1/56 (1.8)	4/75 (5.3)	4/18 (22.2)	0.006	5/131 (3.8)	0.002
ICU admission, *n* (%)	0/56 (0.0)	0/75 (0.0)	0/18 (0.0)	NA	0/131 (0.0)	NA
Need for mechanical ventilation, *n* (%)	0/56 (0.0)	0/75 (0.0)	0/18 (0.0)	NA	0/131 (0.0)	NA

ICU, intensive care unit; MP, *Mycoplasma pneumoniae*; NA, not applicable; SD, standard deviation. * *p* value represents comparisons between the good response, slow response, and no response or progression groups. # *p* value represents comparisons between responder group (a combination of the good response and slow response groups) and non-responder (no response or progression group) group.

**Table 3:** Comparisons of laboratory findings at the time of admission according to treatment responses in *Mycoplasma pneumoniae* pneumonia.

Variables	Good Response Group	Slow Response Group	No Response or Progression Group	*p* Value *	Responder Group	*p* Value #
WBC, ×10^3^/μL	7700 ± 3260	10,090 ± 4810	11,030 ± 5940	0.003	9065 ± 4366	0.138
Neutrophil (%)	60.4 ± 14.0	63.6 ± 14.4	68.8 ± 17.5	0.097	62.2 ± 14.3	0.023
Lymphocyte (%)	26.8 ± 11.0	26.4 ± 12.9	17.9 ± 7.8	0.014	26.6 ± 12.1	0.002
Eosinophil (%)	2.1 ± 2.5	1.8 ± 2.5	1.3 ± 1.9	0.407	1.9 ± 2.5	0.171
Monocyte (%)	9.7 ± 4.4	7.8 ± 3.1	7.6 ± 3.1	0.006	8.6 ± 3.8	0.222
CRP, mg/dL	3.1 ± 3.9	2.5 ± 3.8	6.7 ± 8.1	0.003	2.8 ± 3.8	0.032
ESR, mm/h	39.5 ± 18.6	34.3 ±20.7	37.1 ± 19.9	0.430	36.5 ± 19.9	0.803
Procalcitonin, ng/dL	0.2 ± 0.3	0.3 ± 0.5	0.3 ± 0.5	0.790	0.3 ± 0.4	0.842
LDH, IU/L	670.9 ± 201.5	816.3 ± 328.2	1129.3 ± 493.0	<0.001	756.8 ± 291.3	0.002
AST, IU/L	39.1 ± 20.0	45.5 ± 41.1	58.6 ± 52.7	0.141	42.7 ±33.8	0.586
ALT, IU/L	28.6 ± 27.2	31.8 ± 35.9	53.1 ± 73.1	0.068	30.4 ± 32.4	0.162
Albumin, g/dL	5.3 ± 1.5	5.4 ± 1.5	5.1 ± 1.7	0.841	5.3 ± 1.5	0.286
IgE, kU/L	321.0 ± 449.6 (*n* = 12)	364.3 ± 395.2 (*n* = 21)	1169.2 ± 1121.8 (*n* = 4)	0.018	321.0 ± 449.6 (*n* = 33)	0.171
MP-specific IgM, index	3.5 ± 0.4	4.7 ± 3.2	6.6 ± 2.8	0.001	4.2 ± 3.3	0.004

AST, aspartate aminotransferase; ALT, alanine aminotransferase; CRP, C-reactive protein; ESR, erythrocyte sedimentation rate; IgE, immunoglobulin E; LDH, lactate dehydrogenase; MP, *Mycoplasma pneumoniae*; WBC, white blood cells. * *p* value represents comparisons between the good response, slow response, and no response or progression groups. # *p* value represents comparisons between responder group (a combination of the good response and slow response groups) and non-responder (no response or progression group) group.

**Table 4:** Comparisons of microbiologic characteristics according to treatment responses in *Mycoplasma pneumoniae* pneumonia.

Variables, *n* (%) or Mean ± SD (Range)	Good Response	Slow Response	No Response or Progression	*p* Value *	Responder Group	*p* Value #
Macrolide sensitivity				0.142		0.915
MSMP	6/54 (11.1)	2/75 (2.7)	1/18 (5.6)		8/129 (6.2)	
MRMP	48/54 (88.9)	73/75 (97.3)	17/18 (94.4)		121/129 (93.8)	
A2063G mutation				0.233		0.559
A2063G mutation (−)	5/56 (8.9)	2/75 (2.7)	0/18 (0.0)		7/131 (5.3)	
A2063G mutation (+)	50/56 (90.9)	73/75 (97.3)	18/18 (100.0)		123/131 (93.9)	
Unknown A2063G mutation	1/56 (1.8)	0/75 (0.0)	0/18 (0.0)		1/131 (0.8)	
A2064G mutation				0.486		0.367
A2064G mutation (−)	50/54 (92.6)	71/75 (94.7)	18/18 (100.0)		24/129 (18.6)	
A2064G mutation (+)	0/54 (0.0)	0/75 (0.0)	0/18 (0.0)		97/129 (75.2)	
Unknown A2064G mutation	4/54 (7.4)	4/75 (5.3)	0/18 (0.0)		8/129 (6.2)	
Co-infection with respiratory viruses	16/54 (29.6)	35/73 (47.9)	11/17 (64.7)	0.019	51/127 (40.2)	0.055
Number of co-infected respiratory viruses	0.5 ± 0.9	0.6 ± 0.7	0.7 ± 0.6	0.478	0.5 ± 0.6	0.171
Adenovirus co-infection	4/56 (7.1)	12/75 (16.0)	3/18 (16.7)	0.280	16/131 (12.2)	0.595
Rhinovirus co-infection	10/56 (17.9)	17/75 (22.7)	8/18 (44.4)	0.067	27/131 (20.6)	0.025

MRMP, macrolide-resistant *Mycoplasma pneumoniae*; MSMP, macrolide-sensitive *Mycoplasma pneumoniae*. * *p* value represents comparisons between the good response, slow response, and no response or progression groups. # *p* value represents comparisons between responder group (a combination of the good response and slow response groups) and non-responder (no response or progression group) group.

**Table 5:** Comparisons of radiologic features at the time of admission according to treatment responses in *Mycoplasma pneumoniae* pneumonia.

Variables	Good Response Group	Slow Response Group	No Response or Progression Group	*p* Value *	Responder Group	*p* Value #
Severity of pneumonia based on chest radiography at the time of admission				<0.001		<0.001
Mild	10/56 (17.9)	6/75 (8.0)	0/18 (0.0)		16/131 (12.2)	
Moderate	41/56 (73.2)	51/75 (68.0)	5/18 (27.8)		92/131 (70.2)	
Severe	5/56 (8.9)	18/75 (24.0)	13/18 (72.2)		23/131 (17.6)	
Characteristics of chest radiography at the time of admission				<0.001		<0.001
Peribronchial infiltration	11/56 (19.6)	22/75 (29.3)	2/18 (11.1)		33/131 (25.2)	
Patchy consolidation	32/56 (27.1)	30/75 (40.0)	3/18 (16.7)		62/131 (47.3)	
Lobar consolidation	8/56 (14.3)	17/75 (22.7)	13/18 (72.2)		25/131 (19.1)	
Diffuse nodular opacity	2/56 (3.6)	1/75 (1.3)	0/18 (0.0)		3/131 (2.3)	
Diffuse infiltration	3/56 (5.4)	5/75 (6.7)	0/18 (0.0)		8/131 (6.1)	
Pleural effusion	7/56 (12.5)	10/75 (13.3)	7/18 (38.9)	0.019	17/131 (13.0)	0.005
Involvement of RML	7/56 (12.5)	24/75 (32.0)	5/18 (27.8)	0.033	31/131 (23.7)	0.702
Involvement of lingular segment	18/56 (32.1)	20/75 (26.7)	6/18 (33.3)	0.739	38/131 (29.0)	0.706

RML, right middle lobe. * *p* value represents comparisons between the good response, slow response, and no response or progression groups. # *p* value represents comparisons between responder group (a combination of the good response and slow response groups) and non-responder (no response or progression group) group.

**Table 6:** Factors associated with the treatment responses in MP pneumonia.

**Variables**	Slow Response *		No Response or Progression *	
	**aOR (95% CIs)**	***p* Value**	**aOR (95% CIs)**	***p* Value**
Severity based on chest radiography at admission				
Mild	Ref.		Ref.	
Moderate	2.845 (0.913–8.869)	0.071	NA	
Severe	10.573 (2.303–48.543)	0.002	NA	
Pleural effusion	1.383 (0.470–4.607)	0.556	5.127 (1.404–18.727)	0.013
Respiratory virus co-infection	2.007 (0.924–4.359)	0.078	4.354 (1.374–13.800)	0.012
LDH, IU/L	1.002 (1.000–1.004)	0.015	1.005 (1.002–1.007)	0.001
MP-specific IgM titer at the time of admission, index	1.114 (0.995–1.247)	0.062	1.309 (1.095–1.564)	0.002
**Variables**	**Non-Responder Group #**			
	**aOR (95% CIs)**		***p* Value**	
Duration between symptom onset and admission, days	1.162 (1.025–1.317)		0.019	
Pleural effusion	4.792 (1.549–14.820)		0.007	
LDH, IU/L	1.002 (1.001–1.004)		<0.001	
MP-specific IgM titer at the time of admission, index	1.280 (1.080–1.518)		0.004	
Oxygen need at the time of admission	7.628 (1.764–32.986)		0.007	
Rhinovirus co-infection	3.283 (1.144–9.418)		0.027	

Adjusted by age and sex. * The good response group was considered as the reference group. # The responder group, which is a combination of the good response and slow response groups, was considered as the reference group. aOR, adjusted odds ratio; CI, confidence interval; LDH, lactate dehydrogenase; MP, *Mycoplasma pneumoniae*; NA, not applicable; ref., reference.

**Table 7:** Sensitivity, specificity, positive predictive value, and negative predictive values for ROC curves in Figure 1.

Figure 1	Sensitivity	Specificity	PPV	NPV
A	77.33	44.23	66.67	57.50
B	87.67	64.00	78.05	78.05
C	64.71	98.00	91.67	89.09
D	88.89	90.70	80.00	95.12
E	27.78	99.20	83.33	90.51
F	23.53	100.00	100.00	89.26

PPV, positive predictive value; NPV, negative predictive value.

## Data Availability

The data presented in this study are available on request from the corresponding author. The data are not publicly available due to restrictions.
